# Visualized Analysis of Heavy Ion Radiotherapy: Development, Barriers and Future Directions

**DOI:** 10.3389/fonc.2021.634913

**Published:** 2021-07-09

**Authors:** Yuanchang Jin, Jingwen Li, Jieyun Li, Na Zhang, Kangle Guo, Qiuning Zhang, Xiaohu Wang, Kehu Yang

**Affiliations:** ^1^ Evidence-Based Social Science Research Center, School of Public Health, Lanzhou University, Lanzhou, China; ^2^ Evidence-Based Medicine Center, School of Basic Medical Sciences, Lanzhou University, Lanzhou, China; ^3^ Heavy Ion Treatment Center, Lanzhou Heavy Ions Hospital, Lanzhou, China; ^4^ Institute of Modern Physics, Chinese Academy of Sciences, Lanzhou, China

**Keywords:** heavy ion radiotherapy, Citespace, visualization research, cancer, radiation

## Abstract

**Background:**

Heavy ion radiotherapy (HIRT) has great advantages as tumor radiotherapy.

**Methods:**

Based on 1,558 literatures from core collections of Web of Science from 1980 to 2020, this study visually analyzes the evolution of HIRT research, and sorts out the hotspots and trends of HIRT research using CiteSpace software.

**Results:**

Research on HIRT has received more extensive attention over the last 40 years. The development of HIRT is not only closely related to radiation and oncology, but also closely related to the development of human society. In terms of citation frequency, “International Journal of Radiation Oncology*Biology*Physics” was the top journal. In terms of influence, “Radiotherapy and Oncology” was the top journal. “Radiation therapy” and “carbon ion radiotherapy” were the two most frequently used keywords in this field.

**Conclusion:**

The evolution of the HIRT research has occurred in approximately three stages, including technological exploration, safety and effectiveness research and technological breakthroughs. Finally, some suggestions for future research are put forward.

## Introduction

With the rapid world population growth and aging, cancer as the leading cause of death in the increasingly prominent position (1). According to statistics from the International Agency for Research on Cancer (IARC), the global cancer incidence and mortality rates are increasing rapidly (2). It is estimated that 22 million new cancer cases and 13 million cancer-related deaths occurring annually by 2030 (3). The main treatment methods for malignant tumors include surgical therapy, chemotherapy, and radiation therapy (4). Among them, heavy ion radiotherapy (HIRT) is one of the important methods of radiotherapy for malignant tumors.

Heavy ions generally refer to the particles with atomic number greater than 2 that are ionized (5). After accelerating, the charged particles deposit energy at the end of the range and form a Bragg peak, with a high linear energy transfer (LET) (6). It provides a new therapeutic method for intractable cancers and radioresistant tumors (7). In the 1970s, Lawrence Berkeley National Laboratory (LBNL) was the first to apply HIRT in clinical trials. However, helium and neon ions were still the mainstream of heavy ion line research in the United States at that time (8). In 1994, Japan’s National Institute of Radiological Science (NIRS) built the world’s first heavy ion medical accelerator in Chiba (HIMAC), which is dedicated to heavy ion cancer treatment and research of radiation medicine (9). In June 1994, the first group of patients received heavy ion beam therapy with HIMAC at NIRS (10). The patients treated included head and neck tumors, brain tumors, lung cancer, liver cancer, prostate cancer and cervical cancer. Since that time, over 20,000 patients have been treated with carbon ion radiation therapy (CIRT) (11). In 1997, Helmholtz Centre of Heavy Ion Research (GSI) in Darmstadt, Germany, achieved the heavy ion beam conformal radiotherapy and real-time on-line monitoring of beam current (6, 12). In 2005, the Institute of Modern Physics (IMP), Chinese Academy of Sciences, based on the heavy ion research facility in Lanzhou (HIRFL), built the heavy ion treatment terminal for superficial tumors, which also made China the fourth country in the world to conduct heavy ion clinical trials.

In recent years, HIRT has become a cutting-edge technology in tumor radiotherapy, and its potential advantages have been continuously explored, and the efficacy of tumor treatment has been further affirmed (13). As a result, the number of publications on HIRT for tumors has increased rapidly. However, the performance, productivity, and impact of these studies are still unknown.

At present, bibliometrics has been recognized as the most active sub-discipline in the international library and information field, and has become the mainstream of information science research, reflecting the trend of quantification of contemporary disciplines (14). Compared with other analysis methods, scientometric analysis is a quantitative analysis method that combines mathematics and statistical methods (15), and is a good choice for evaluating the trend of research activities (16). In addition, scientometric analysis focuses on the measurement characteristics of research literature in a certain field, which helps researchers grasp the development characteristics of this field and guide follow-up work (17). This study systematically evaluated the research on HIRT that was included in the WOSCC from database built to the end of August 2020. This review was conducted to address the following research questions:

Q1: What are the overall Scientometric data extracted from HIRT research literature?

Q2: What are the recent and emergent trends and issues in HIRT research?

Q3: What promising future research directions are suggested based on the recent empirical findings in HIRT learning?

## Material And Methods

### Source of Data

The literature data used in this study was downloaded from the Science Citation Index Extension (SCIE) and Social Science Citation Index (SSCI) databases of Web of Science. SCIE and SSCI are the most frequently used databases in bibliometric analysis. These two databases cover more scientific and authoritative publications than other databases. In addition, SCIE and SSCI provide literature citation information, keywords and reference information. The search time is set to “all year”, and the search formula was set to TS = “carbon beam therap*” OR “carbon ion beam radiation therap*” OR “carbon ion beam radiotherapy*” OR “carbon ion beam therap*” OR “carbon ion radiation therap*” OR “carbon ion radiotherap*” OR “carbon ion therap*” OR “carbon ion treatment*” OR “heavy ion radiation therap*” OR “heavy ion radiotherap*” OR “heavy ion therap*” OR “heavy ion treatment*”.

### Inclusion Criteria

We included articles and reviews published in different academic journals. Letters, editorial materials, Meeting abstracts, conference presentations, book reviews, news items, and corrections were excluded. The language was limited to English, without specifying species restrictions.

### Research Methods

Co-citation is an important part of citation analysis. If two papers are cited by one or more subsequent papers at the same time, the two papers are considered to have the co-citation relationship (18). Because the co-citation analysis method is scientific and objective, its analysis objects have been extended from papers to authors, disciplines and journals. These three types of co-citation are all based on the co-citation of the paper. Journal co-citation means that the documents of two journals are cited by the documents of other journals at the same time (19). The number of documents of other journals that meet the conditions is the co-citation strength of the journal. Journal co-citation organically links many journals that seem to have no external connections, thus revealing the interdependence and cross-over relationship between journals (20). Using journal co-citation relationships can determine the professional limits and content coverage of certain journals, reveal the development status, structure and interrelationships between journals, and confirm the core journals of the subject.

Keywords are the core summary of a paper. Analysis of keywords in the paper can give a glimpse of the topic of the article. The idea of keyword co-occurrence analysis comes from the concept of citation coupling and co-citation in bibliometrics (21). That is, when two keywords that can express the research theme or research direction of a certain subject field appear in the same document, it indicates that there is a certain internal relationship between the two words. And the more times it appears, the closer the relationship and the closer the distance. Counting the frequency of the two pairs of subject words in the same document can form a common word network composed of these word pairs. Using factor analysis, cluster analysis and multidimensional scale analysis and other multivariate statistical methods, the research hotspots, structure and paradigm of the subject can be summarized (22).

CiteSpace is designed as a progressive knowledge domain visualization tool (23). In this article, we used CiteSpace to analyze the evolution, knowledge structure, hot issues and development trends of the heavy ion research field from 1980 to the end of August 2020, carried out multi-dimensional network analysis and draw the corresponding knowledge map. In this study, CiteSpace was used (1) for journal distribution analysis (2), for keyword co-occurrence analysis, and (3) for reference and keyword burst analysis.

## Results

### Annual Publishing Trends

**Figure 1:**
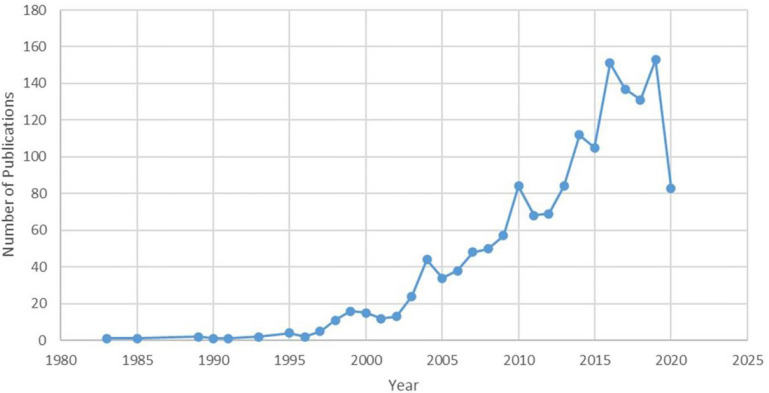
Publication of HIRT related papers (as of the end of August 2020).

A total of 1,558 related articles were included for visual analysis. The overall trend of publications increased from one publication in 1983 to 153 publications in 2019 (**Figure 1**). At 1980s, research on HIRT was still in its infancy, with a small amount of related publications; by 1996, the annual publication amount was less than five. Subsequently, Japan and Germany successively used carbon ions as the beam for tumor treatment to conduct clinical trial research. Therefore, from 1997 to 2008, the research on HIRT increased steadily, and the number of peer-reviewed papers increased from 5 to 50. Since 2009, the number of literatures on heavy ion radiotherapy has increased rapidly, which has received more attention than before. It may be due to the remarkable curative effect of HIRT in Japan HIMAC, especially twice cure rate for liver cancer and lung cancer (24, 25), which has conquered the medical community of all countries.

### Distribution of Journals

CiteSpace has a dual-map analysis module, which can display citation trajectories, knowledge flow and the distribution of papers in other information fields through dual-map overlay analysis of journals, and uses Blondl algorithm to form journal clusters (26). On the dual-map overlay analysis result of the journal, the left side shows the journal distribution of the citing literature and the cited literatures is on the right. The curve is the citation line, which shows the whole sequence of citations. The size of the ellipse shows the number of papers and authors published in the journal. The more papers published in the journal, the longer the vertical axis of the ellipse; the more authors, the longer the horizontal axis of the ellipse.

There were four citation paths. The first orange path, papers published in molecular/biology/immunology journals mostly cited journals in molecular/biology/genetics area; the next orange path, papers published in molecular/biology/immunology journals partially cited journals in health/nursing/medicine area; the first green path, papers published in medicine/medical/clinical journals partially cited journals in molecular/biology/genetics area, the bottom green path, papers published in medicine/medical/clinical journals partially cited journals in health/nursing/medicine area.

**Figure 2:**
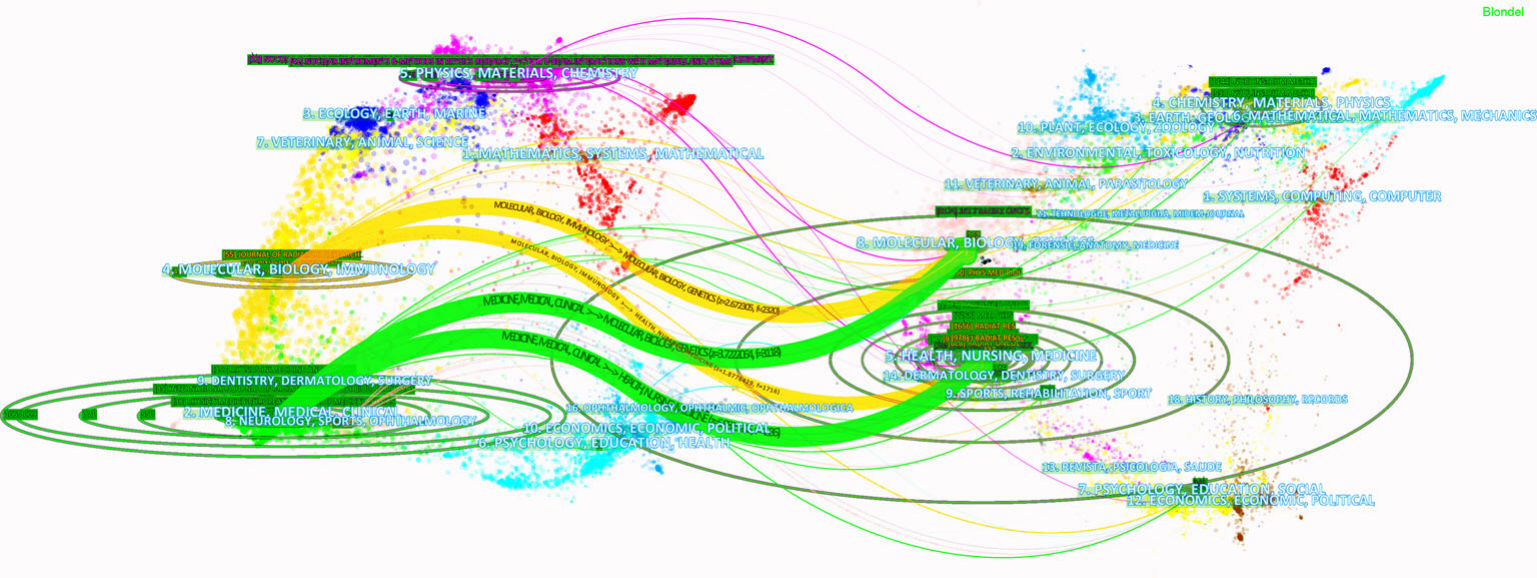
Dual-map overlay of HIRT literature.

In the field of HIRT, the research mainly cited two disciplines as the research foundation (the right side of **Figure 2**): health/nursing/medicine and molecular/biology/genetics. The most frequently published journals in these two disciplines are “International Journal of Radiation Oncology*Biology*Physics” and “Clinical Cancer Research”. Their related research results have been applied to medicine/medical/clinical and molecular/biology/immunology (the left side of **Figure 2**). “Physics in Medicine & Biology” and “Journal of radiation research” are the most published journals in these two disciplines. In addition, some journals in the fields of psychology, pedagogy, economics, politics, society, history, philosophy, sports, etc. are also cited. It shows that the development of HIRT is not only closely related to radiation and oncology, but also closely related to the development of human society.

### Co-Citation Analysis of Journals

**Figure 3:**
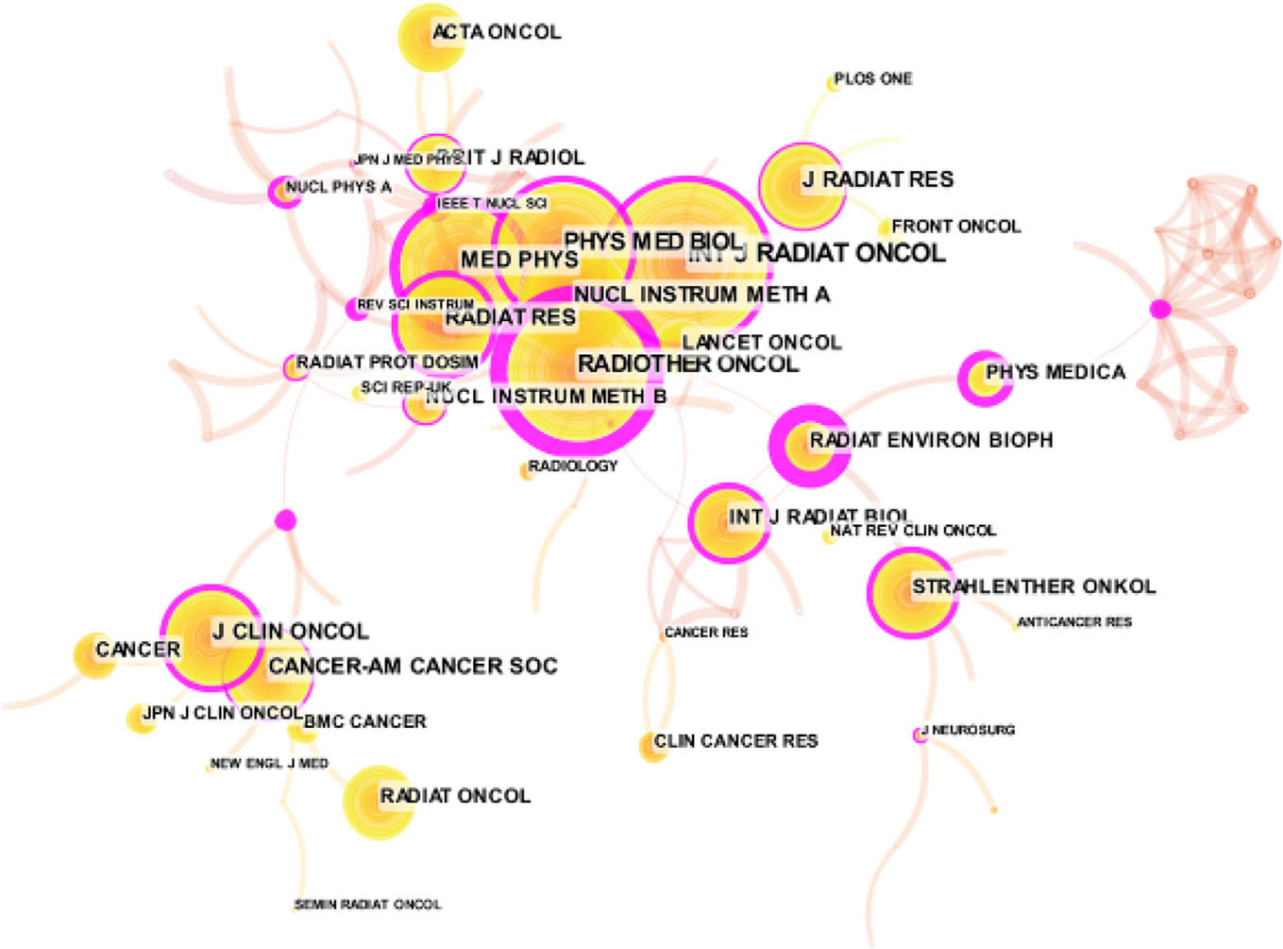
Co-cited analysis of journals related to HIRT literature.

Citespace was used to analyze the co-citation of journals (**Figure 3**). The co-citation of journal analysis shows the distribution of important knowledge sources in a field (27). Because the academic influence of a journal mainly depends on the frequency of its citation. In Citespace, we selected the node type as “Cited Journal,” extracted the “Top20” with the highest frequency in each time slice. Finally, we obtained a co-citation network consisting of 86 nodes and 320 connections (**Figure 3**). As we have noticed, the larger the node, the higher the citation frequency. At the same time, through the co citation frequency analysis of core journals, we can effectively reveal the publication quality level of a certain journal. “International Journal of Radiation Oncology*Biology*Physics” have been cited 1,186 times, ranking first, followed by “Radiotherapy and Oncology” (913), “Physics in Medicine & Biology” (794), “Medical Physics” (629).

Centrality is used to indicate the importance of nodes. It is usually shown as a purple ring in the figure. The thickness of the ring reflects the importance of intermediateness. The greater the thickness, the higher the centrality of the node, and the higher the importance of the node. In **Figure 3**, the purple ring of “Radiotherapy and Oncology” is the thickest, followed by “Radiation and Environmental Biophysics” and “Review of Scientific Instruments”. The centrality values are 0.97, 0.94, and 0.58, respectively. Except for “Radiotherapy and Oncology”, we found that the frequency of citations is not proportional to centrality. Even if the frequency of citations is higher, it does not necessarily mean that the journal has greater influence.

### Keywords Co-Occurrence Analysis

**Figure 4:**
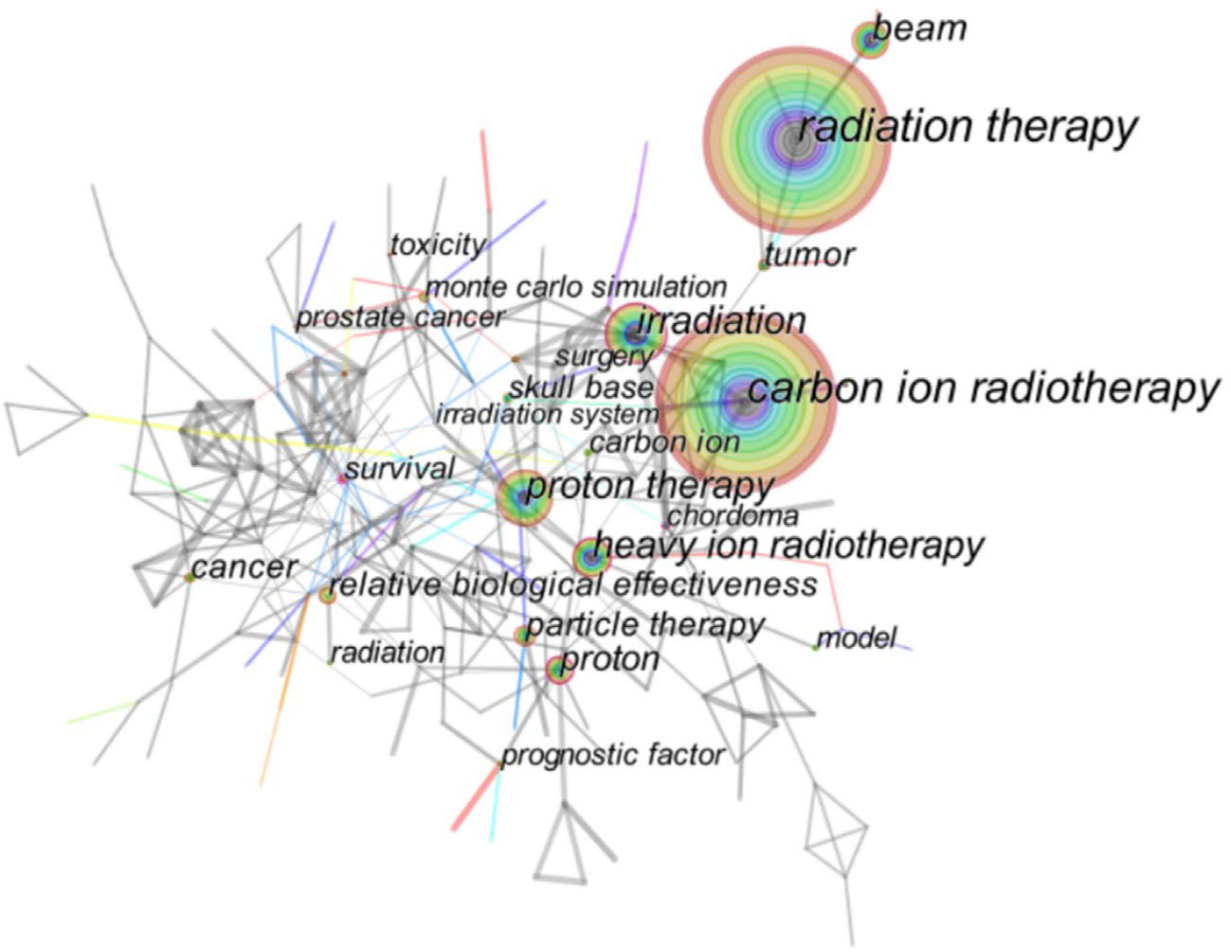
Co-ocurrence analysis of keywords related to HIRT literature.

Keywords can directly and accurately reflect the theme of the article (28). Co-occurrence analysis of keywords through CiteSpace can intuitively understand the research hotspots in this field. **Figure 4** shows the keyword co-occurrence map in HIRT domain. The larger the node, the more times the keyword appears, and the stronger the relevance with the topic of the paper. In this part, we described the distribution of a keyword network consisting of 225 nodes and 929 connections. The top keywords are “radiation therapy” (676), “carbon ion radiotherapy” (664), followed by “irradiation” (262), “proton therapy” (250), and “heavy ion radiotherapy” (220). Under this theme, it is not surprising that other high-frequency keywords appear, except for “proton therapy”. Proton and heavy ion beam therapy is the most advanced radiotherapy technology recognized by the international community. Both protons and heavy ions are charged particles (29). Unlike conventional rays such as X-rays, gamma rays, and electron rays, protons and heavy ions with a certain energy have a Bragg peak that concentrates the deposited energy after entering human tissues. In the treatment of tumors, the energy of protons (or heavy ions) can be adjusted and the Spread Out Bragg Peak (SOBP) can be used to make the rays act on tumors of different depths and sizes (30). In this way, high-dose multi-field irradiation of the tumor target area can be achieved, and at the same time, the normal tissues around the tumor can be exposed to as little radiation damage as possible. The structural composition principle of the medical heavy ion accelerator system and the medical proton accelerator system is basically the same (31). At present, some compact medical proton/heavy ion accelerators that have been built or are under construction in the world have the functions of both proton and heavy ion radiotherapy. Therefore, it is not surprising that “proton therapy” appears frequently as a keyword in this field.

The distribution of keywords shows a diversified trend, and the research topics are divided into the following categories: HIRT technology, such as Monte Carlo simulation, dose escalation, treatment planning, pencil beam scanning, relative biological effectiveness, etc.; various tumors, such as advanced adenoid cystic carcinoma, small-lung cancer, hepatocellular carcinoma, prostate cancer, squamous cell carcinoma, soft tissue sarcoma, etc.; outcome indicators such as toxicity, complications, survival, mortality, efficiency.

## Research Frontiers And Challenges

**Figure 5:**
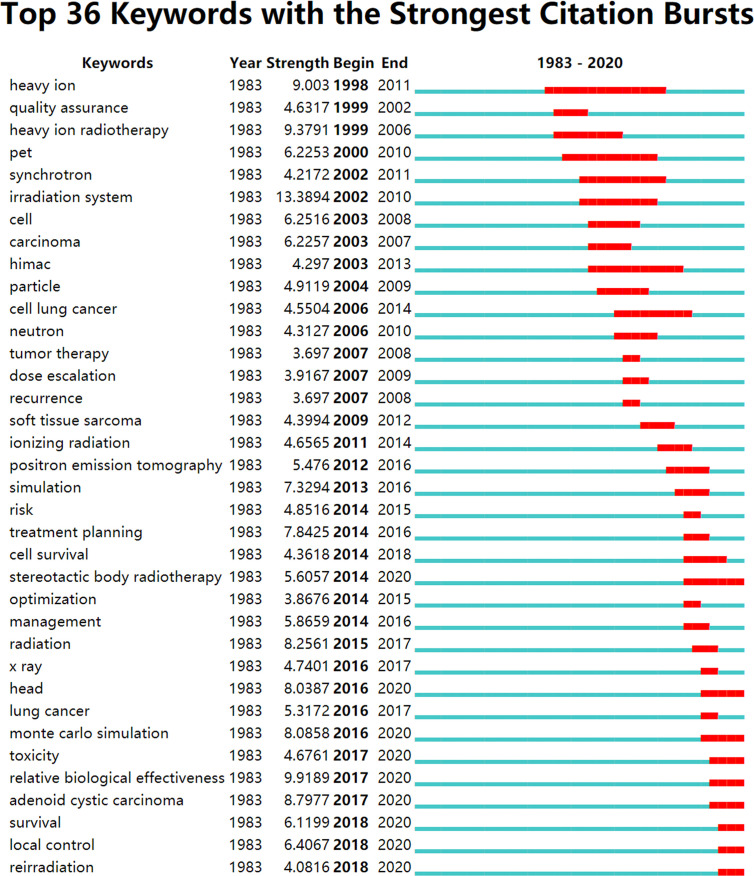
Burst keywords of HIRT-related research.

**Figure 6:**
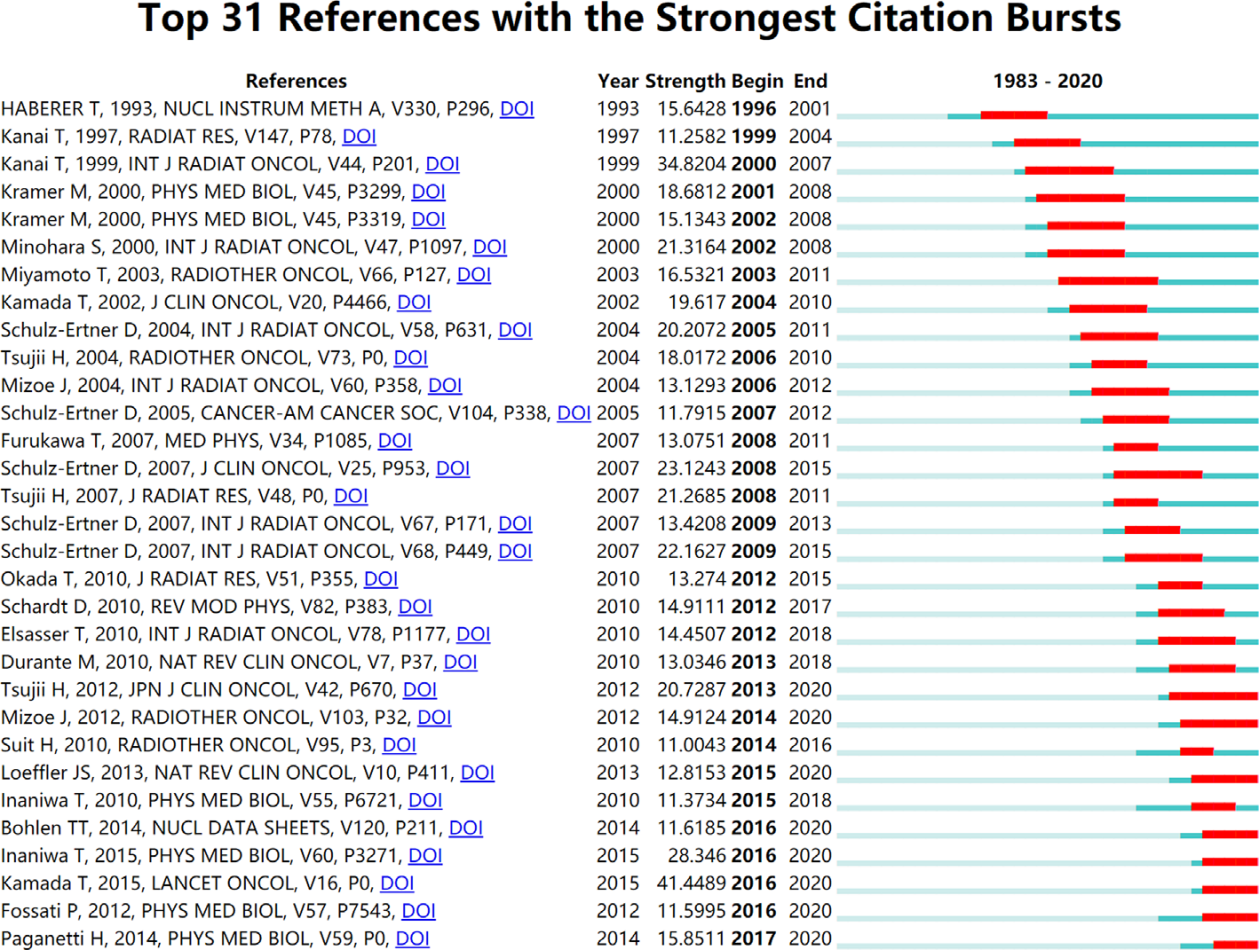
Burst articles of HIRT-related research.

Burst terms are regarded as indicators of the frontiers of research within a period of time, which appear due to trends and sudden changes in a certain period of time (32). The burst terms have two key aspects: burst strength and duration. The former represents the burst intensity, and the latter includes the beginning and end of the burst time, as shown by the red line in **Figures 5** and **6**.

The first stage (1993–2002): In this stage, the frontier of HIRT mainly focused on the research of technology exploration. Electron linear accelerator is the core component of HIRT technology (33). In terms of structure and principle, medical heavy ion accelerator system and medical proton accelerator system are basically the same. It mainly includes accelerator system, beam transmission system, treatment terminal system and treatment planning system (TPS). The accelerator system is the core part of medical accelerators (34). At present, there are basically three types of accelerators used in medical heavy ion accelerator treatment centers in the world: linear accelerator, cyclotron and synchrotron (35). The main accelerators of the existing or under construction medical heavy ion accelerator treatment centers all use adjustable-energy synchrotrons, and the injectors use linear accelerators or cyclotrons. For example, HIMAC and GSI both use linear accelerators. However, the injector of the medical heavy ion accelerator built by HIRFL, Chinese Academy of Sciences, uses cyclotron with reliable operation and smaller footprint.

In the treatment planning system, the accuracy of dose calculation is particularly important. The main drawback of general radiotherapy is that the normal tissues surrounding the tumor will also be exposed to high doses. Radiation injury to the normal tissues can cause serious complications. In order to reduce complications, it is necessary to reduce the radiation dose, which results in insufficient tumor dose and limits the improvement of therapeutic efficacy.

The energy change of the “Bragg peak” produced by heavy ions is generally only a few millimeters wide. Many tumors that can be treated clinically are larger than a few millimeters in diameter. Only by superimposing multiple “Bragg Peaks” can the tumor be covered (36). With the help of pencil beam scanning technology, the energy can be changed and multiple Bragg peaks can be superimposed to achieve more accurate and accurate treatment. Pencil beam scanning technology is the key technology of proton and heavy ion therapy (37). The tumor is simulated layered by electronic computer, and then the rays are controlled to scan point by point and layer by layer, so as to improve the accuracy of radiation exposure and the treatment effect.

While heavy ion treatment of tumors has significant advantages, it will also encounter technical difficulties. Lung cancer, liver cancer, pancreatic cancer, these tumors will be slightly displaced with breathing exercise. In order to confirm that the tumor in motion enters the “window” of irradiation, and the scanning can complete the whole dose coverage in a short time, the authoritative experts of particle therapy used the respiratory gating technology to solve the problem. Respiratory gating technology uses advanced respiratory motion baroreceptors to transmit the signal to the computer, and the computer outputs the signal to the synchrotron to control the synchrotron to turn on and off the rays (38). Respiratory gating technology effectively reduces the radiation volume of radiation on normal organs, and the curative effect is more accurate.

In summary, the biological basis of radiotherapy such as protons and photons is the “4R theory” commonly used in radiobiology: repair, reoxygenation, redistribution, repopulation (39). The “4R theory” is the basic biological theory of traditional radiotherapy, which determines that proton, photon and other radiotherapy need to increase the number of fractional irradiations to reduce side effects and enhance curative effects. The biological effects of carbon ion rays go beyond the above categories. Its ability to kill tumor cells has little to do with the oxygen concentration and periodic distribution of tumor cells. Using CIRT, the number of divisions is less (even one time can be completed), and the damage to normal tissues is small, but the killing effect on cancer cells is greatly improved (40).

The second stage (2003–2014): Research on the effectiveness and safety of HIRT has become a hot topic in the field of tumor radiotherapy. Carbon ion is the most used heavy ion for treatment. According to the data particle therapy co-operative group (PTCOG), as of the end of 2019, 12 particle therapy centers around the world can implement CIRT. By the end of 2019, a total of 34,138 patients have been treated, including more than 29,000 cancer patients by NIRS and more than 4,000 patients by GSI.

In 2015, NIRS published a study in Lancet Oncology to introduce the experience in the past 20 years on CIRT for cancer treatment (41). It has been proved that carbon ions are effective in the treatment of radiation resistant head and neck tumors. For example, the 5-year overall survival (OS), progression free survival (PFS) and local control (LC) were 74, 44 and 68%, respectively. The 5-year local control rate and survival rate can reach 88 and 86% in patients with inoperable sacral chordoma treated with carbon ion. However, it should be noted that there are 15/95 patients with sciatic nerve injury. The survival rate of patients with stage I peripheral non-small cell lung cancer treated with carbon ion is similar to the best survival result of photon stereotactic body radiation therapy (SBRT).

Compared with photon, proton and other radiotherapies, HIRT shows obvious clinical advantages.

Victor et al. (42) conducted a systematic review of nine studies on CIRT of 632 cases of skull base chordoma and chondrosarcoma. The results showed that in the chordoma-only study, the estimated 1-year, 5-year, and 10-year LC were 99, 80, and 56%, respectively, and in the chondrosarcoma-only study, 99, 89, and 88%, respectively. In the chordoma-only study, the 1-year, 5-year, and 10-year OS were 100, 94, and 78%, respectively. In the chondrosarcoma study alone, the 1-year, 5-year, and 10-year overall survival probabilities were 99, 95, and 79%, respectively. The incidence of early and late toxicity (grade 2/3) in all study groups ranged from 0 to 4%. CIRT treatment of skull base chordoma and chondrosarcoma has promise in terms of tumor control, overall survival rate and early and late toxicity risk. Zhang et al. (43) compared the effectiveness of CIRT, proton radiotherapy (PRT), and photon-based intensity modulated radiotherapy (IMRT) in the treatment of malignant sinus tumors. Through cross-group analysis, OS (75.1%) after CIRT was significantly higher than PRT (66.2%) or IMRT (63.8%). After CIRT, LC (80.2%) was significantly higher than PRT (72.9%) or IMRT (67.8%). However, for OS and LC, there is no significant difference between PRT and IMRT. CIRT provides better OS and LC for patients with malignant tumors of the nasal cavity and paranasal sinuses. Kong et al. (44) compared the recent adverse effects of CIRT and intensity-modulated X-ray therapy (IMXT) in the treatment of recurrent nasopharyngeal carcinoma. The prescribed dose of CIRT was 50–60 Gy E (2.0–2.5 Gy E each time), and the prescribed dose of IMXT was 56–66 Gy E (2.0–2.1 Gy E each time). The results showed that the recent adverse reactions of CIRT are far less than IMXT.

Concurrent radiotherapy and chemotherapy is currently the main treatment for inoperable locally advanced non-small-cell lung cancer (NSCLC), but many patients cannot tolerate it. Janneke et al. (45) conducted a systematic review of qualified studies of CRT, SBRT, simultaneous radiotherapy (CCR), PRT and CIRT for NSCLC. The results showed that the 2-year OS of stage 1 inoperable NSCLC ranged from 53% for CRT to 74% for CIRT. The 5-year OS of CRT (20%) is significantly lower than that of SBRT (42%), PRT (40%) and CIRT (42%). It is concluded that the survival rate of particle therapy is higher than that of CRT.

A phase I/II dose gradient escalation clinical trial by Yamada et al. (46) studied the efficacy of CIRT in the treatment of 186 patients with locally recurring rectal cancer, with a total dose of 67.2–73.6 GyE/16 times/4 weeks. The results of the second phase trial showed that the 5-year LC and OS of patients with a total dose of 73.6 GyE were 88 and 59%, respectively, and no >3 grade adverse reactions were found. NIRS conducted a phase I/II clinical trial of preoperative carbon ion radiotherapy for resectable esophageal cancer from 2004 to 2008 (47). In addition to one patient with acute respiratory distress syndrome and the therapeutic relationship uncertain, the other patients were not uncontrollable adverse reactions. After follow-up observation, 11 of the 31 patients relapsed. The cause of the recurrence was considered to be related to lymph node metastasis.

Although the clinical application of CIRT is still in exploration, more and more clinical trials are reported to support its remarkable curative effect, especially in refractory tumors, radiation-resistant tumors and complex tumors, and it is expected to significantly shorten the treatment time.

The third stage (2015–2020): In this stage, the research focus of HIRT was technological breakthrough. High RBE is the most significant feature of heavy ion in biology. It requires much less dose than conventional radiation to achieve the same killing effect on tumor cells. RBE of proton and photon is about 1–1.1 (48). According to the different doses and the observed biological effect endpoints, the RBE of carbon ions is generally between 1.5 and 4.5 (49). HIRT can place the tumor in the Bragg peak with high dose and high biological effect. The normal tissue in front of the target is in the range of low dose and low LET, and the damage is minimal. The normal tissues behind the target are irradiated with low dose. DNA is the most important target of radiation on cells. Through direct ionization, the carbon ion beam causes multiple lethal damage to the DNA duplex and kills cancer cells completely (50). The proton and photon rays generally play a role through indirect ionization to produce free radical injury, which leads to sublethal damage to DNA single-strand breaks, which can easily cause tumor recurrence (51).

Furthermore, heavy ions also have a strong killing effect on hypoxic cancer cells that are not sensitive to conventional radiation. When exposed to low LET rays, the radiation sensitivity of hypoxic cells decreases significantly (52). But when the LET of heavy ions exceeds 200 keV/μm, there is almost no oxygen effect. The lethal effect of heavy ions on cells is hardly affected by the cell cycle. In different cell cycle, the radiosensitivity of low LET is different, but for heavy ion beam, the radiosensitivity of high LET has little fluctuation (53).

In the past 20 years, based on the clinical dose system defined by radiobiology, tens of thousands of patients have received CIRT for various tumors in NIRS. Through clinical experience, including extensive dose escalation studies, an optimal dose division plan has been established for each tumor, which can be regarded as the standard for CIRT.

At present, there are two methods to calculate the dose distribution of human tissue. One is the analytical dose calculation algorithm, which mainly includes the pencil beam algorithm and the wide beam algorithm. The other is Monte Carlo (MC) dose algorithm, which uses particle transport software to simulate actual heavy ion beam treatment conditions and calculate the radiation dose of human tissue (54). Due to its high accuracy and simple simulation process, the MC dose algorithm has become an algorithm under development in the current TPS.

The MC dose algorithm can accurately model the complex problems (complex geometry, complex physical processes, complex radioactive source arrangements, etc.) involved in radiotherapy, while using less approximation (55). With the substantial increase in computer processing speed, the continuous reduction in computational cost and the introduction of variance reduction techniques, a variety of MC software has been developed and applied in the field of medical physics (56).

In 2011, NIRS began to use pencil beam scanning (a new beam delivery method) for clinical treatment, and used this opportunity to update the clinical dose system. The requirement of the updated system is to correct the oversimplification in the original system and coordinate with the original system to maintain the established dose fractionation plan. In the updated system, the radiation quality of the therapeutic carbon ion beam was obtained through MC simulation, and its biological effectiveness was predicted through the theoretical model. Both systems provide a uniform clinical dose distribution within the target range consistent with the prescription. Under all test conditions, the average physical dose provided by the updated system to the target is consistent with the dose provided by the original system within ±1.5%. The updated system reflects the physical and biological characteristics of the therapeutic carbon ion beam more accurately than the original system. At the same time, it is allowed to continue to use the dose fractionation scheme established by the original system on the near-infrared spectrometer (57).

In summary, heavy ions have more advantages in biological effects. It is beneficial to treat tumors that are not sensitive to photon rays; and the heavy ion treatment tumor dose distribution is better, which is beneficial to increase tumor dose and reduce normal tissue damage. How to accelerate the calculation speed while maintaining the high-precision characteristics is the main subject of the MC dose calculation method. The development of faster and more accurate dose calculation methods is a hot research topic in the future.

The main obstacle to the current development of HIRT: Although HIRT has achieved encouraging clinical effects, the current development of HIRT for tumors is relatively slow. In addition to capital, insurance and other market factors, we should also see the constraints of technological development: ① The better choices for beam types are uncertain. At present, the beam currents used in the treatment of cancer with heavy ion beam is carbon ion beam. Whether carbon ion beam is the best beam current for clinical use is still worthy of discussion. In particular, it is very important to carry out research on the biological effects of nitrogen, oxygen, and fluorine for selecting the best beam for the next generation of heavy ion beams for cancer treatment. ② The safety research of HIRT is still insufficient. Some studies have found that heavy ions have a carcinogenic risk. At the same time, heavy ion treatment of tumors will have some long-term health risks. At present, researches on improving the safety of HIRT for tumors and ensuring the quality of life of patients after rehabilitation are crucial. ③ The further promotion of this emerging technology of HIRT is still facing many problems. First of all, with the construction and development of heavy ion treatment centers, the number of patients receiving heavy ions is increasing, so the demand for professionals is particularly tense. At the same time, the complexity of the heavy ion therapy accelerator device limits its promotion. Finally, high construction, operation and maintenance costs have also greatly restricted related research and development.

*Future development:* As a frontier hotspot of medical research, CIRT for malignant tumors has incomparable advantages over traditional radiotherapy, including precise dose distribution, powerful tumor cell lethality and the monitorability of carbon ion beams. Making full use of the above-mentioned physical and biological advantages of carbon ions in the treatment of malignant tumors can produce a series of clinical advantages such as good therapeutic effects, light adverse reactions, and accurate positioning. However, for different treatment goals, there may be better choices for beam types. From the perspective of technology promotion, helium ion beam therapy may be more promising. The cell experiment results showed that the RBE of helium ions is higher than that of carbon ions and neon ions, but OER is smaller than that of carbon ions and neon ions. In terms of accelerator technical requirements and return on investment, helium ions have a smaller mass than carbon and oxygen, and require less beam energy to reach the same depth in the body. Therefore, the requirements for accelerators are low and the investment cost is low. Helium ion accelerators are easy to achieve miniaturization. At present, research institutions and companies have begun to pay attention to helium ion cancer treatment technology. Besides, oxygen ion beam is not only the basic element of human body like carbon ion beam, but also can be used for feedback tracking with PET. Compared with carbon ion beam, oxygen ion beam has less nuclear fragment yield and smaller lateral scattering. More importantly, theoretically, it is speculated that oxygen ion beam is likely to have smaller OER, stronger lethal effect on cancer stem cells and better clinical effect than carbon ion beam, which is likely to be the best beam current for cancer treatment by heavy ion beam. Therefore, we need to strengthen the study of the biological effects of oxygen ion beams on tumor cells (especially cancer stem cells).

Achieving more miniaturization of medical accelerator systems and reducing treatment costs will be the focus of future equipment research. Since the medical heavy-ion accelerator system and the medical proton accelerator system are basically the same in structure and composition principle, the functions of proton beam and heavy-ion beam radiotherapy are integrated into the same medical accelerator system, so as to achieve the purpose of compound treatment, comprehensive utilization and cost reduction. At present, some compact medical proton/heavy ion accelerators that have been built or are under construction in the world have both proton and heavy ion radiotherapy functions, such as the Japan Hyogo Ion Beam Medical Center (HIBMC), Heidelberger Ionenstrahl-Therapiezentrum (HIT), the Italian National Centre for Oncological Hadrontherapy (CNAO), and Shanghai Proton and Heavy Ion Hospital in China (58). Although the use of advanced technologies such as superconductivity in recent years has made medical proton/heavy ion accelerators more compact, there is still a large distance compared with the more miniaturized equipment that people expect. The demand for high therapeutic gain, miniaturization and low cost promotes the continuous advancement of medical proton accelerator and medical heavy ion accelerator technology (59).

When promoting any new treatment method, ensuring safety is the top priority, especially for expensive and extremely complex heavy ion facilities. The theories of 4R in photon fractionation therapy are not fully applicable to HIRT (39). It needs to be improved based on the experience of photon radiotherapy. Heavy ion-induced NDA cluster damage is difficult to repair, and the repair rate is low. It is the main cause of death, aberration and even carcinogenesis. Animal experiments show that the risk of lung cancer (60), liver cancer (61) induced by low-dose heavy ions is much higher than that of photon, and there are health risks such as secondary cancer (62). It is of great significance to establish a reasonable experimental model and carry out in-depth biological research to improve the safety of heavy ion therapy for tumor and ensure the quality of life of patients after rehabilitation (63).

## Conclusions

This study provides historical insights into the trends of HIRT research. The number of published papers significantly increased over the last 40 years, and the overall trend of publications increased from one publication in 1980 to 153 publications in 2019. The trends and focus of applied HIRT research were highlighted. In addition to the molecular, biology, immunology, medicine, that HIRT research has traditionally belonged to, in recent years, some journals have published HIRT-related research in the fields of psychology, pedagogy, economics, politics and so on. In terms of citation frequency, “International Journal of Radiation Oncology*Biology*Physics” was the top journal. In terms of influence, “Radiotherapy and Oncology” was the top journal. “radiation therapy” and “carbon ion radiotherapy” were the two most frequently used keywords in this field. Technological breakthrough in HIRT field was the latest frontier.

Although this is the first bibliometric study in HIRT research, several limitations should be addressed. The electronic database is limited to Web of Science, and other electronic databases are not searched and analyzed, for example, PubMed, Embase, Cochrane Library. Furthermore, the non-English papers were excluded. In addition, we selected several keywords and tried to expand the search using topic patterns (64). In fact, no search criteria are 100% perfect, and a few publications may not be included in our search. In addition, the parameter settings of the CiteSpace software are very complicated, and different time slices or different threshold settings will also affect the research results to a certain extent. Nevertheless, the bibliometric method and Cite Space visual analysis provide a reliable perspective for us to study the research hotspots and frontier issues in a certain field (32). However, the software has certain limitations. The data sources that can be analyzed by CiteSpace mainly come from the Web of Science database, and the analysis capabilities for documents from other database sources are limited (65). Furthermore, due to the limitation of the Web of Science database, the results of this study may be biased. And the hotspot analysis only analyzes the emerging literatures, which cannot fully reflect the situation of the research hotspots it represents.

In the future, ① For different therapeutic targets, the best beam should be selected. ② Achieving more miniaturization of medical accelerator systems and reducing treatment costs will be the focus of future equipment research. ③ Establishing a reasonable experimental model and carrying out in-depth biological research are of great significance to the safety research of HIRT.

## Author Contributions

Conception and design: KhY, YcJ, and JwL. Professional content guidance: QnZ and XhW Search and collection of data: JyL, NZ, and KlG. Data analysis and interpretation: JyL, NZ, and KlG; Manuscript writing: YJ and JwL. All authors contributed to the article and approved the submitted version.

## Funding

This work was supported by the 2017 Lanzhou Talent Innovation and Entrepreneurship Project Funding, Key Technologies for Basic and Clincal Application of Domestic Heavy Ion Accelerator for Tumor Treatment (Grant no.2017-RC-23), The Science and Technology Support Program of Gansu Provincial Science and Technology Department (Grant no.[2016]1604FKCA109), Health Industry Scientific Research Program of Gansu Province in 2019 (Grant no. GSWSKY-2019-91), and The Fundamental Research Funds for the Central Universities (Grant no. 18LZUJBWZX006 and Grant no. 2019jbkyzy002): Evidence-based Social Science Research.

## Conflict of Interest

The authors declare that the research was conducted in the absence of any commercial or financial relationships that could be construed as a potential conflict of interest.
